# Engineering of PA-IIL lectin from *Pseudomonas aeruginosa *– Unravelling the role of the specificity loop for sugar preference

**DOI:** 10.1186/1472-6807-7-36

**Published:** 2007-06-01

**Authors:** Jan Adam, Martina Pokorná, Charles Sabin, Edward P Mitchell, Anne Imberty, Michaela Wimmerová

**Affiliations:** 1 National Centre for Biomolecular Research, Faculty of Science, Masaryk University, Kotlarska 2, 611 37 Brno, Czech Republic; 2 CERMAV-CNRS (affiliated with Université Joseph Fourier), Grenoble BP53, F-38041 Grenoble cedex 09, France; 3 E.S.R.F., Experiments Division, BP-220, F-38043 Grenoble cedex 09, France; 4 Institute of Biochemistry, Faculty of Science, Masaryk University, Kotlarska 2, 611 37 Brno, Czech Republic

## Abstract

**Background:**

Lectins are proteins of non-immune origin capable of binding saccharide structures with high specificity and affinity. Considering the high encoding capacity of oligosaccharides, this makes lectins important for adhesion and recognition. The present study is devoted to the PA-IIL lectin from *Pseudomonas aeruginosa*, an opportunistic human pathogen capable of causing lethal complications in cystic fibrosis patients. The lectin may play an important role in the process of virulence, recognizing specific saccharide structures and subsequently allowing the bacteria to adhere to the host cells. It displays high values of affinity towards monosaccharides, especially fucose – a feature caused by unusual binding mode, where two calcium ions participate in the interaction with saccharide. Investigating and understanding the nature of lectin-saccharide interactions holds a great potential of use in the field of drug design, namely the targeting and delivery of active compounds to the proper site of action.

**Results:**

*In vitro *site-directed mutagenesis of the PA-IIL lectin yielded three single point mutants that were investigated both structurally (by X-ray crystallography) and functionally (by isothermal titration calorimetry). The mutated amino acids (22–23–24 triad) belong to the so-called specificity binding loop responsible for the monosaccharide specificity of the lectin. The mutation of the amino acids resulted in changes to the thermodynamic behaviour of the mutants and subsequently in their relative preference towards monosaccharides. Correlation of the measured data with X-ray structures provided the molecular basis for rationalizing the affinity changes. The mutations either prevent certain interactions to be formed or allow formation of new interactions – both of afore mentioned have strong effects on the saccharide preferences.

**Conclusion:**

Mutagenesis of amino acids forming the specificity binding loop allowed identification of one amino acid that is crucial for definition of the lectin sugar preference. Altering specificity loop amino acids causes changes in saccharide-binding preferences of lectins derived from PA-IIL, via creation or blocking possible binding interactions. This finding opens a gate towards protein engineering and subsequent protein design to refine the desired binding properties and preferences, an approach that could have strong potential for drug design.

## Background

Carbohydrates are essential for life and in addition to their classical role as structural and energy storage components, they play crucial roles in many recognition events, signalling and communication processes. Recognition of glycoconjugates is an important event in biological systems and is frequently provided through protein-carbohydrate interactions. Many pathogens exploit host cell-surface glycoconjugates as receptors for attachment, tissue colonization and/or invasion. This is related to the fact that the structure of saccharides offers a wide range of diversity for encoding information, a fact directly tied to the variability of possible isomeric configurations of monosaccharides, increased even further when taking into account the possible linkages between the sugar units [1].

The recently heavily investigated area of protein-saccharide interactions holds a great potential of use in the field of drug design, namely targeting and delivery of active compounds to the proper site of action [2]. Another therapeutical area involving lectin-saccharide interaction is the inhibition of bioadhesion, since bacteria are often able to effectively adhere to the surface membranes of the host cells via lectin binding, thus enabling subsequent colonization and advancement of the disease.

*Pseudomonas aeruginosa *is a gram negative bacterium and an opportunistic pathogen that infects immunocompromised patients and which is often involved in nosocomial infections [3]. The bacterium is also responsible for chronic respiratory tract infections of individuals under mechanical ventilation and patients suffering from cystic fibrosis (CF). Carbohydrates play an important role in infections, since they are a target for bacterial binding through virulence factors such as adhesins, present on pili or flagella, and soluble lectins [4]. In the particular case of CF patient lungs, an increase of fucosylation is observed, both at the levels of epithelia glycoconjugates [5] and altered mucins [6], and a fucose binding lectin from *P. aeruginosa *(PA-IIL) has been demonstrated to bind specifically to CF airline culture cells [7]. The tetrameric lectin that was first identified and characterised from the *P. aeruginosa *cytoplasm [3] has been shown to be present in large quantities on the outer membrane of the bacteria and to be also involved in biofilm formation [8]. Recently, it has been proposed that the lectin role lies in pilus genesis and proteolytic activity [9].

Through protein crystallography at high resolution, a precise three dimensional knowledge of the lectin has allowed the structural basis of the interaction between PA-IIL and fucose and fucose containing oligosaccharides to be understood [10,11]. An unusual binding mode, mediated by two calcium ions, is responsible for the micromolar affinity for fucose which is much higher than that classically observed in protein-carbohydrate interactions [12]. Previous inhibition tests have demonstrated that other monosaccharides are also recognised by PA-IIL with affinity in the decreasing order of L-fucose (Fuc) > L-galactose (L-Gal) > D-arabinose (Ara) > D-fructose (Fru) > D-mannose (Man) [13]. Indeed, these monosaccharides all have either an L-galacto or a D-manno configuration that present two equatorial and one axial hydroxyl groups when in the ^1^C_4 _and ^4^C_1 _pyranose conformation, respectively. This particular stereochemistry is a requirement for the coordination of the two calcium ions as observed in the X-ray crystal structures of PA-IIL complexed with various monosaccharides [10,12,14,15]. Precise equilibrium association constants, together with their enthalpic and entropic contributions, were evaluated for PA-IIL interacting with these monosaccharides and the crystal structures have been determined at very high resolution for the complexes with monosaccharides [10,14].

**Figure 1:**
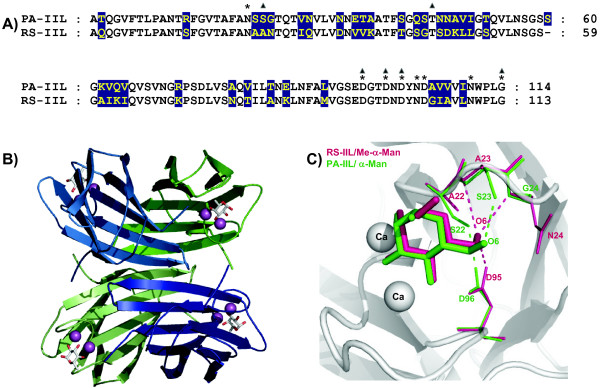
**Comparison of PA-IIL lectin from *Pseudomonas aeruginosa *with its homolog RS-IIL lectin from *Ralstonia solanacearum***. A) Alignment of amino acid sequences of PA-IIL and RS-IIL. The differing amino acids are in yellow with blue background, the amino acids participating in saccharide binding are marked by triangles, and amino acids participating in calcium coordination are marked by stars. B) Graphical representation of the overall structure of PA-IIL tetramer (ribbon) complexed with L-fucose (sticks) and calcium ions (cpk). PDB code 1UZV [10]. C) Superposition of binding sites of RS-IIL/Me-α-Man (structure 1UQX [16]) and PA-IIL/Man (mannose moiety of the trimannose in structure 1OVS [15]) with crucial amino acids highlighted. In PA-IIL, the hydroxyl group of Ser22 makes the hydrogen bond with Asp96 and O6 of mannose interacts with Ser23. In RS-IIL, Asp96 makes directly hydrogen bond with of O6 of methyl mannoside.

In 2004, the structure of RS-IIL, an ortholog of PA-IIL present in the phytopathogenic bacterium *Ralstonia solanacearum*, was determined [16]. Structures of both proteins show similarity, but they display a different order of affinity towards monosaccharides. RS-IIL has a preference for mannose, whereas PA-IIL is the fucose-binding lectin. A comparison of amino acid sequences of PA-IIL and RS-IIL indicates that only one amino acid at position 23 is different amongst those involved in calcium or sugar binding. The structural comparison of PA-IIL and RS-IIL complexed with mannose revealed the importance of a three amino acid motif centred at residue 23 that influences the fine specificity of the lectins (Figure 1).

This work presented here focuses on defining the fine specificity of PA-IIL and the influence of individual amino acids in the binding site for mannose/fucose which contribute to the lectin's preference. Considering the importance of the specificity loop, mutants of PA-IIL were designed. These mutants were constructed by replacing each of the amino acids of the so-called specificity loop, i.e. amino acids in positions 22, 23 and 24 for those present in RS-IIL (i.e, constructing mutants named S22A, S23A and G24N). Mutagenesis was followed by structural and thermodynamic studies to probe the importance of the specificity loop composition.

## Results

### Expression and purification of recombinant proteins

**Figure 2:**
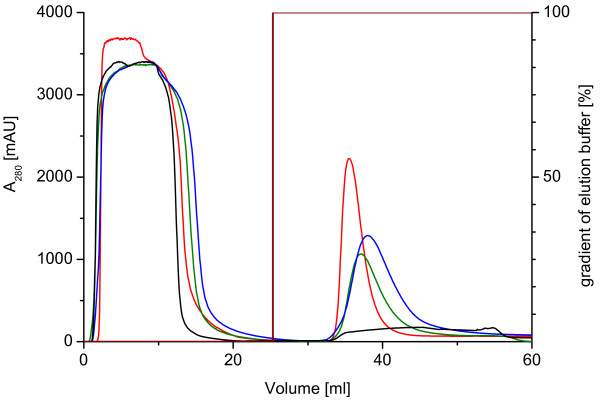
**Purification of mutants by affinity chromatography**. Comparison of chromatograms from purification of PA-IIL mutants S22A (black), S23A (blue), G24N (green) and PA-IIL wild type (red) by affinity chromatography on Mannose agarose column (HR 10/10). Loading buffer: 20 mM Tris/HCl, 100 mM NaCl, 100 μM CaCl_2_, pH 7.5; Elution buffer: 20 mM Tris/HCl, 100 mM NaCl, 100 μM CaCl_2_, 0.1 M D-mannose, pH 7.5; Sample: cytoplasmic soluble protein fractions.

The lectin mutants S22A, S23A, G24N and PA-IIL wild type were prepared following the procedure as described in the Methods section. A different behaviour in the elution of the S22A mutant was observed during elution from a mannose agarose column. Whilst the other mutants and wild-type protein are eluted from the column as a sharp protein peak using 0.1 M D-mannose, S22A showed very wide and smeared peak during chromatography (Figure 2). A typical yield of about 30 to 50 mg of purified proteins per litre of culture was obtained during each cultivation.

### Crystal structure of mutants and their complexes with monosaccharides

Mutants of PA-IIL were first co-crystallised with fucose and then soaked with the methyl-derivative of fucose and mannose. This procedure allowed high resolution data sets to be obtained, diffracting from 1.3 to 1.7 Å resolutions depending on the mutant. All crystal structures belong to the P2_1 _space group except for the S23A mutant that crystallizes in space group C2 (Table 1). In all cases, the asymmetric unit contains one copy of the biological PA-IIL tetramer.

**Table 1:** Data collection and refinement statistics

	**PA-IIL G24N**		**PA-IIL S22A**		**PA-IIL S23A**
	*Me-β-D-Man*	*Me-α-L-Fuc*	*Me-β-D-Man*	*Me-α-L-Fuc*	*Me-α-L-Fuc*
*Data collection*					
Beamline (ESRF)	ID29	ID14-2	ID29	ID14-2	ID14-2
Wavelength (Å)	0.987	0.933	0.976	0.933	0.933
Resolution range (Å)	1.70–31.13	1.50–28.69	1.30–21.26	1.70–34.69	1.30–16.07
Highest resolution shell (Å)	1.70–1.79	1.50–1.54	1.30–1.33	1.70–1.79	1.30–1.37
Space Group	P2_1_	P2_1_	P2_1_	P2_1_	C2
Cell dimensions	a = 50.4 Å; b = 80.1 Å; c = 52.5 Å; β = 109.6°	a= 52.6 Å; b = 74.9Å; c = 53.5 Å; β = 114.7°	a = 52.5 Å; b = 73.0 Å; c = 54.7 Å; β = 94.3°	a= 52.7 Å; b = 73.1Å; c = 54.9 Å; β = 94.3°	a = 95.1 Å; b = 45.7 Å; c = 88.0 Å; β = 94.2°
Matthews Coef (Å^3^/DA)	2.13	2.04	2.22	2.25	2.03
Solvent content (%)	42.2	39.7	44.7	45.3	39.4
Measured/unique reflections	146877/42206	224211/60074	421970/99982	160284/43407	292549/88919
Average multiplicity^1^	3.5 (3.0)	3.7 (3.7)	4.2 (3.8)	3.7 (3.6)	3.3 (3.2)
Completeness^1 ^(%)	97.8 (88.7)	99.7 (99.7)	99.2 (96.4)	95.4 (92.4)	95.8 (93.0)
Average^1 ^*I/σI*	20.1 (6.6)	14.8 (3.3)	23.1 (12.4)	14.4 (3.5)	16.2 (2.6)
R merge^1,2 ^(%)	0.054 (0.20)	0.060 (0.37)	0.042 (0.08)	0.085 (0.32)	0.054 (0.35)
Wilson *B*-factor (Å^2^)	9.68	12.31	10.28	11.29	12.86
*Refinement*					
R_crys_^3 ^(No. observations)	0.143 (40067)	0.151 (57022)	0.139 (94963)	0.177 (41187)	0.170 (84498)
R_free_^4 ^(No. observations)	0.172 (2117)	0.193 (3024)	0.155 (4993)	0.223 (2200)	0.197 (4421)
*Total number of atoms*: Hetero/Protein/solvent	60/3414/445	75/3460/535	68/3499/750	65/3361/524	56/3584/592
*Number of side chains modelled with alternative conformations*	12	16	24	7	34
*Average refined B-factors per chains *(A/B/C/D/water)	7.8/9.2/81/7.4/22.0	10.2/9.7/10.2/10.2/26.4	6.9/7.4/7.3/7.2/23.8	9.0/9.2/9.3/10.8/22.3	10.8/10.9/9.8/11.3/26.2
*RMS deviation from ideality*					
Bonds (Å)	0.013	0.016	0.011	0.011	0.013
Angles (°)	1.26	1.58	1.42	1.29	1.47
Number of outliers on Ramachadran plot	0	0	0	0	0
PDB deposition code	2JDY	2JDU	2JDN	2JDM	2JDP

^1 ^Numbers in parentheses refer to the highest resolution shell.^2^R_merge _= Σ|I - ⟨ I ⟩|/Σ⟨I⟩, Where I = observed intensity.^3^R_cryst _= Σ||Fo - Fc||/Σ |Fo|, where |Fo| = observed structure factor amplitude and |Fc| = calculated structure factor amplitude.^4^R_free _is R_cryst _for 3 % of the reflections excluded from the refinement

**Figure 3:**
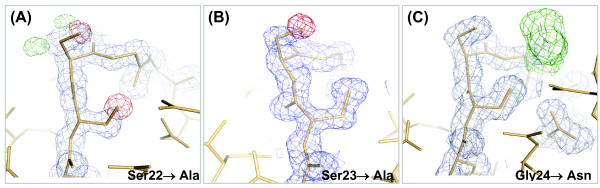
**Difference electron density maps at mutated residues**. Electron density maps *2fo-fc *(contoured at 1.0 sigma, in blue) and *fo-fc *(contoured at 3.0 sigma in green and -3.0 sigma in red) around the specificity binding loop amino acids 22–23–24, generated after molecular replacement with the structure of native wild type protein. (A) S22A complexed with Me-α-Man (with alternative conformations of Ser23), (B) S23A complexed with Me-α-Fuc, (C) G24N complexed with Me-α-Man.

Difference maps created after molecular replacement of PA-IIL mutant data using the native protein as the search probe clearly show excess and/or absence of electron density at the positions of mutated residues (Figure 3). The overall structures of the PA-IIL mutants retain the properties of the wild type protein complexed with fucose, whose structure has been previously described [10,12] (Figure 1B). Briefly, the structure is tetrameric with each monomer characterized by a binding site containing two calcium ions and a saccharide. The site is made up primarily by one long β-hairpin loop with the participation of a shorter loop and of the C-terminal acidic group at Gly114 of the neighbouring monomer. The two calcium ions are very close to each other, with distances that vary from 2.73 to 2.75 Å in the four monomers.

Initial refinement of the crystal structures revealed that during soaking, the monosaccharides of interest had not always replaced the fucose molecules originally present in the protein complexes. Hence, amongst the four monomers that form the asymmetric unit, substitution of the sugar occurred in the four monomers of PA-IIL S23A/Me-α-Fuc and G24N/Me-α-Man, but only in three monomers of the S22A/Me-α-Man and S22A/Me-α-Fuc complexes and in only two monomers of G24N/Me-α-Fuc. The other two monomers of G24N/Me-α-Fuc are lacking both calcium ions and don't contain any sugar moiety in the binding site. Substitution of fucose by the mannoside was not successful at all during soaking crystals of PA-IILS23A/fucose in the Me-α-Man solution and the structure was not further refined.

Altogether five crystal structures have been refined, corresponding to the complexes of mutants S22A, S23A and G24N with Me-α-Fuc and S22A and G24N with Me-α-Man. Refinement statistics are summarised in Table 1. The sugar ring orientations of Me-α-Fuc and Me-α-Man in the binding site of mutants are similar to those observed in complexes with the wild type lectin [10,15].

### Structure of complexes of PA-IIL mutants and methyl α-L-fucopyranoside

In the binding site of all three mutants, the O2, O3 and O4 atoms of the methyl fucoside participate in the coordination spheres of the calcium ions, O3 occupying the space between the ions and interacting with both of them, whereas the O2 and O4 interact each with only one of the ions. The hydroxyl groups of the fucoside are involved in creating hydrogen bonds with the surrounding amino acids with O3 creating a bond to the side chain oxygen of Asp 99 and O4 to the carboxyl oxygen from Gly 114 belonging to the neighbouring monomer. These interactions are omnipresent in all the investigated mutants, as is the hydrophobic interaction between the methyl groups of the fucoside and of Thr 45.

**Figure 4:**
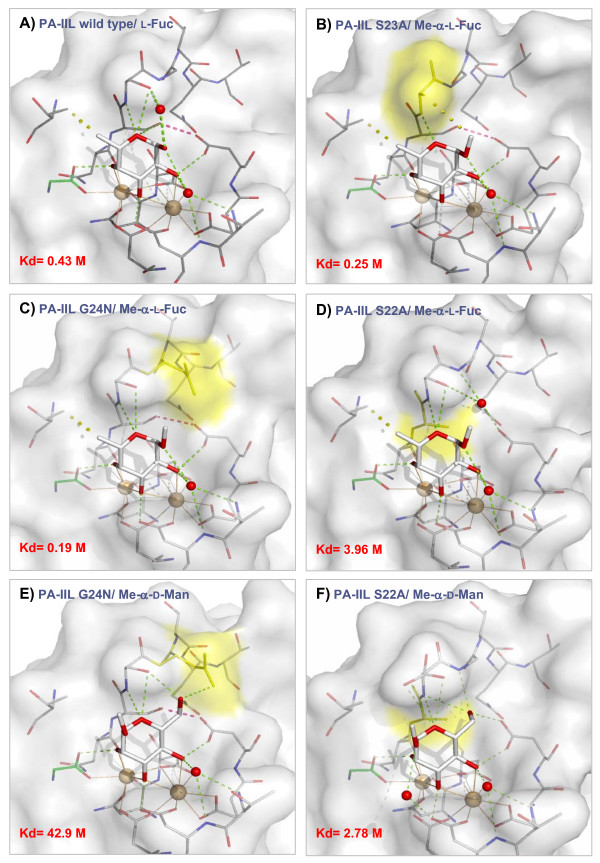
**Crystal structures of PA-IIL mutants**. Detail of binding sites of PA-IILwild type/L-fucose (PDB code 1GZT [12]) and PA-IIL mutants complexed with Me-α-Fuc and Me-α-Man. Mutated amino acids are in yellow, water molecules are represented as red balls. Calcium ions and corresponding coordination bonds are in beige. Hydrogen bonds and hydrophobic interactions are represented by green and yellow dashed lines, respectively. The crucial interaction between Ser22 and Asp96 is shown as dashed red lines.

Some interactions vary depending on the nature of the mutated residues. In case of serine at position 22, the hydroxyl group of the serine side chain is able to make a hydrogen bond with the carboxyl group of Asp96. When this serine is mutated to alanine, the hydroxyl group at 22 is absent and the side chain of Asp96 interacts with a water molecule. In case of mutation of Ser23 to Ala23, the original hydrogen bond between Ser22 and Asp96 is retained. A new hydrophobic interaction is enabled between the methyl group at O1 of the fucoside and the side chain of Ala23. In the case of the G24N mutation, no new interaction is observed (Figure 4A–D).

### Structure of complexes of PA-IIL mutants and methyl α-D-mannopyranoside

In accordance with the crystal structure of PA-IIL/man complex [15], the mannoside binds into the different mutated lectins via coordination of O2, O3 and O4 with the calcium ions, i.e. in the opposite orientation to that observed for fucose. The position of the fucoside C5 methyl moiety is occupied by the O1 hydroxyl group of the mannoside, and the O6 hydroxymethyl group replaces the fucoside O1 hydroxyl group. In the crystal structure with wild type PA-IIL, the O6 atom points out of the binding site and establishes a hydrogen bond to the side chain of Ser 23. The same orientation is observed in the crystal structure of the mutant G24N, with an additional hydrogen bond formed with the side chain of Asn24 (Figure 4E) In the S22A mutant, the same O6 adopts a totally different orientation since it points towards the floor of the binding site, in the location that was occupied by the side chain of Ser22 in the wild type. In this position, it can establish a strong hydrogen bond with the carboxyl group of Asp96 (Figure 4F). Table 2 summarises contact distances between mutated lectins and sugar ligands.

**Table 2:** Comparison of contact distances (in Angstroms) in binding sites of the PA-IIL mutants

	**PA-IIL G24N**		**PA-IIL S22A**		**PA-IIL S23A**
	Me-β-D-Man	Me-α-L-Fuc	Me-β-D-Man	Me-α-L-Fuc	Me-α-L-Fuc
Man (O6) -- Ser23 (OG)	2.80	-	3.50	-	-
Man (O6) -- Asn24 (ND2)	3.31	--	-	-	-
Man (O6) -- Asp96 (OD2)	-	-	2.67	-	-
Man (O6) -- Ser23 (N)	-	-	3.20	-	-
Man (O6) -- Gly24 (N)	-	-	2.94	-	-
O5--Ser22 (OG)	-	3.44	-	-	3.44
O5--Ser23 (OG)	3.20	3.19	3.19	3.35	-
Asp96 (OD2) -- Ser22 (OG)	2.71	2.78	-	-	2.70
Asp96 (OD2) -- H_2_O (2)	-	-	-	2.60	-
H_2_O (2) -- Ser23 (OG)	-	-	-	-	-
Fuc (O1) -- H_2_O (1)	-	3.08	-	3.19	3.03

### Thermodynamics of interactions of PA-IIL and its binding to monosaccharides

**Figure 5:**
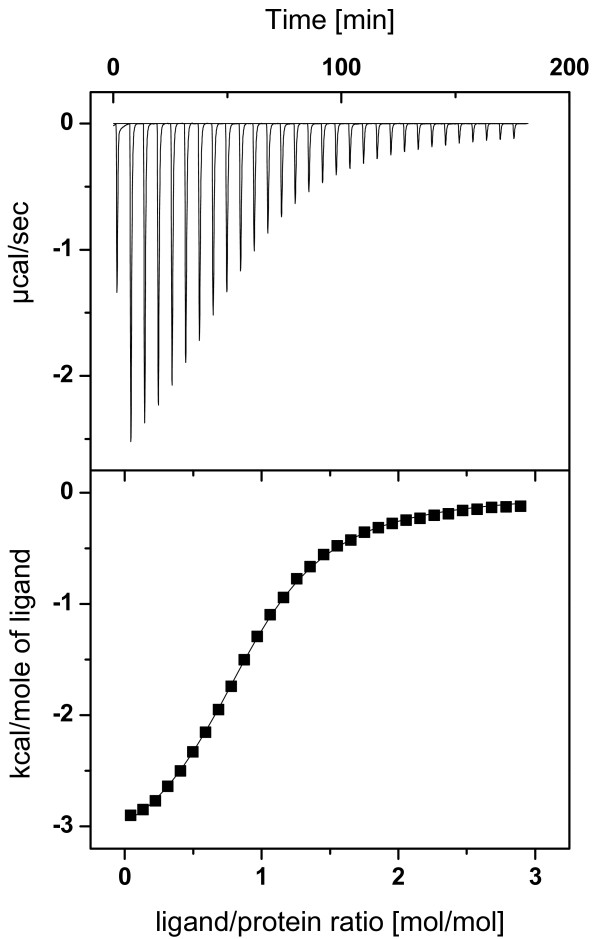
**Isothermal titration microcalorimetry**. Titration microcalorimetry results for binding of Me-α-Man (3.804 mM) to S23A (295 μM) in 0.1 M Tris buffer (pH = 7.5) with 30 μM CaCl_2_. A) Data obtained from 29 automatic injections (10 μL) of Me-α-Man each into the S23A-containing cell. B)Plot of the total heat released as a function of ligand/protein molar ratio for the titration shown in panel A. The solid represents the best least-squares fit for the obtained data.

Thermodynamic data were recorded for the interaction of PA-IIL and its mutants with methyl glycosides of the monosaccharides of interest, i.e. Me-α-Fuc, Me-α-Man and also with Me-α-L-Gal, for comparison. The use of methyl derivatives of the saccharides avoids the problems related to anomeric disorder in solution and moreover gives a better mimic of real biological interactions where the monosaccharides are not free but linked to glycoconjugates. The result of a typical titration calorimetry measurement is shown in Figure 5. The results exhibited a monotonic decrease in the exothermic heat of the binding until saturation is achieved. The values of equilibrium association constant K_a_, binding enthalpy ΔH, and stoichiometry per monomer *n *were obtained by fitting the classical equation for single-site binding [17]. The other thermodynamic parameters, free energy, and entropy of binding (ΔG and ΔS) were calculated as described in the Methods. The thermodynamic characteristics of the interaction of PA-IIL and its mutants are summarized in Table 3.

**Table 3:** Thermodynamics of lectin/sugar binding.  Thermodynamics of binding for different monosaccharides interacting with PA-IIL and its mutants, compared to thermodynamic characteristics of mannose-preferring lectin RS-IIL from *Ralstonia solanacearum *as determined by ITC at 25°C. Standard deviations calculated from three independent measurements for all ligands.

	K_A _[×10^4^M^-1^]	K_D _[μM]	ΔG [kJ/mol]	ΔH [kJ/mol]	-TΔS [kJ/mol]
**Me-α-L-Fuc**					
G24N	524 (± 36.7)	0.19 (± 0.03)	-38.4 (± 0.2)	-40.1 (± 0.7)	1.8 (± 0.7)
S23A	418 (± 72.8)	0.25 (± 0.01)	-37.8 (± 0.4)	-33.9 (± 1.2)	-3.8 (± 0.9)
**PA-IIL**^**a**^	**235 (± 8.0)**	**0.43 (± 0.01)**	**-36.4 (± 0.1)**	**-41.3 (± 1.0)**	**4.9 (± 1.0)**
S22A	26.4 (± 4.9)	3.96 (± 0.91)	-30.9 (± 0.5)	-37.1 (± 0.6)	6.2 (± 0.9)
RS-IIL^b^	< 1	> 100			
**L-Fuc**					
S23A	23.8 (± 3.4)	4.3 (± 0.6)	-30.6 (± 0.4)	-26.9 (± 0.2)	-3.7 (± 0.6)
G24N	15.2 (± 2.3)	6.6 (± 0.9)	-30.0 (± 0.4)	-31.1 (± 0.4)	1.1 (± 0.7)
**PA-IIL**^**c**^	**12.0 (± 1.0)**	**8.3 (± 0.7)**	**-29.0 (± 0.1)**	**-27.7 (± 0.5)**	**-1.3 (± 0.5)**
S22A	10.3 (± 0.4)	9.7 (± 0.4)	-28.6 (± 0.1)	-26.8 (± 0.7)	-1.8 (± 0.6)
RS-IIL	nd	nd	nd	nd	nd
**Me-α-L-Gal**					
**PA-IIL**^**a**^	**85.0 (± 5.0)**	**1.17 (± 0.07)**	**-33.8 (± 0.1)**	**-31.5 (± 0.6)**	**-2.3 (± 0.7)**
G24N	82.2 (± 18.8)	1.29 (± 0.35)	-33.7 (± 0.6)	-18.7 (± 0.3)	-15.0 (± 0.9)
S23A	81.6 (± 14.4)	1.23 (± 0.07)	-33.9 (± 0.4)	-26.8 (± 1.7)	7.1 (± 1.3)
S22A	6.75 (± 0.31)	15.0 (± 0.66)	-27.6 (± 0.5)	-19.2 (± 0.2)	-8.3 (± 0.3)
RS-IIL	nd	nd	nd	nd	nd
**Me-β-D-Man**					
RS-IIL	438 (± 2.0)	0.23 (± 0.01)	-37.9 (± 0.1)	-24.9 (± 0.2)	-13.0 (± 0.2)
S22A	36.0 (± 1.8)	2.78 (± 0.14)	-31.7 (± 0.1)	-24.3 (± 0.6)	-7.4 (± 0.7)
G24N	2.34 (± 0.17)	42.9 (± 3.12)	-24.9 (± 0.2)	-27.1 (± 0.8)	2.1 (± 0.9)
S23A	1.91 (± 0.33)	54.0 (± 9.32)	-24.4 (± 0.4)	-16.7 (± 2.3)	-7.7 (± 2.7)
**PA-IIL**^**a**^	**1.42 (± 0.05)**	**71.0 (± 3.00)**	**-23.7 (± 0.1)**	**-17.8 (± 1.3)**	**-5.9 (± 0.3)**

^a ^taken from Sabin, C. *et al*, 2006 [14]

^b ^value < 10^4 ^M^-1 ^[18]

^c ^taken from Perret, S. *et al*, 2005 [11]

As expected, the stoichiometry of binding was always close to four molecules of saccharide for one tetrameric unit of the lectin. For some protein batches, binding stoichiometry was around 3 signalling a possible deactivation of one subunit. Nevertheless, the other binding characteristics were not significantly affected by this and data evaluation clearly showed that all binding sites are equal and independent to each other.

The observed values of association constants for **Me-α-Fuc **varied as a function of the specificity loop mutation. The mutants S23A and G24N show approximately 200% and 160% higher affinity compared to PA-IIL, whose dissociation constant equals 430 nM. The S22A mutant displays a significant drop to a mere 11% of the PA-IIL affinity, its dissociation constant being consequently almost ten fold higher (3.96μM). Still, it is a considerably larger affinity than is displayed for the binding of Me-α-Fuc by the mannose-preferring lectin RS-IIL (the "non-detectable" value is lower than 1.10^-4 ^M).

The results for non-methylated **fucose **have shown that the absence of methyl group at the anomeric position leads to an affinity decreased around one order of magnitude in the case of PA-IIL and mutants S23A and G24N. This effect is a general observation in protein-carbohydrate interactions. For S22A, the affinity drop was only slight, changing from 3.96 μM to 9.7 μM in terms of dissociation constant.

For **Me-α-L-Gal**, the affinity of the PA-IIL is approximately four times lower than in the case of Me-α-Fuc, with a dissociation constant of 1.17 μM [14]. Comparing the affinity of the mutants towards Me-α-Gal, the mutants S23A and G24N displayed almost the same level of affinity as the wild type protein, with dissociation constants of 1.23 and 1.29 μM, respectively, whilst the mutant S22A has a considerably lower affinity of less than one tenth of it. The affinity towards methyl galactoside is generally lower than to methyl fucoside.

The situation is different when it comes to **Me-α-Man**. Overall affinity towards this monosaccharide is considerably lower than the previous ones. The values of dissociation constants are one hundred times higher than in the case of methyl fucoside, again comparable for the wild type and mutants S23A and G24N. On the other hand, the affinity towards mannoside increased approximately thirty times for the mutant S22A. Nevertheless, even for the S22A mutant, the affinity value is approximately ten times lower than for the mannose-preferring lectin RS-IIL.

## Discussion

In general, interactions between proteins and carbohydrates present a balance of hydrogen bonds and hydrophobic contacts. PA-IIL is a rather unique case since its interaction with fucose is established mainly through coordination by calcium ions and hydrogen bonds. The only hydrophobic interaction is established between the methyl group at C5 of fucose and the side chain of Thr45. The contribution of functional groups at the C5 position of fucose was already discussed in a previous article showing that hydrophobic interactions are not crucial for sugar binding [14]. The relative contributions of the different thermodynamic parameters are more difficult to rationalise. The entropic term is a sum of multiple effects. A favourable entropic contribution is obtained when the increase of solvent entropy coming from water driven out of the site is correlated with minimal loss of conformational degrees of freedom in either ligand or protein chains during binding. Protein-carbohydrate interactions cannot usually meet these criteria because of the strong entropy penalty usually observed. Interestingly, the study of the sugar free PA-IIL structure has shown the presence of three water molecules in the binding site, strongly coordinated to the calcium ions [15]. These water molecules are replaced by three hydroxyl groups in the complex with saccharide. The release of the water molecules, that are bound more strongly than in a classical carbohydrate binding site, is the reason of aforementioned behaviour and entropy terms closed to zero.

### Complexes of mutants with methyl α-L-fucopyranoside

Previous work regarding the comparison of PA-IIL and RS-IIL has hinted that the reason behind the different affinities may be in the composition of the specificity binding loop. These observations are further confirmed by the results of the current experiments that point to a very special role for Ser22. In the absence of the side-chain hydroxyl group (mutant S22A), there is no hydrogen bond to Asp96 and the available carboxyl group of Asp96 binds a water molecule. This is in accordance with the observed entropy penalty.

Mutant S23A is even more interesting. The affinity towards methyl fucoside is almost two times higher than in the case of the native protein. Moreover, there is a significant difference in the contribution of both components of Gibbs energy. The native protein displays an unfavourable entropic contribution TΔS of -4.9 kJ/mol, whereas the mutant shows a favourable entropic part of 3.8kJ/mol, making the total difference 8.7kJ/mol. The hydroxyl group of Ser23 in the native protein interacts with the fucoside O5, creating an unfavourable entropy contribution. If this interaction was absent, the entropic contribution would become favourable for the free energy and consequentially, for the affinity. By replacing Ser23 with alanine, another interaction is also generated: the hydrophobic interaction between the side chain of alanine and the C5 methyl group of fucose. The sugar free structure of PA-IIL shows some flexibility of Ser23 which becomes specifically oriented during sugar binding and increases the contribution to the entropy penalty once sugar is bound. A comparison of thermodynamic data of all lectins from the PA-IIL family lacking serine at position 23, (S23A, RS-IIL and fucose-preferring lectin CV-IIL from *Chromobacterium violaceum *that was described recently [18]) shows a total positive entropy contribution to binding in all cases. This could imply that the presence of amino acids in the binding site, which don't lose their flexibility during binding of a sugar moiety and are not crucial for the interaction can favourable influence the entropy term. Findings relating to the residue at position 23 may be very useful in further mutagenesis and lectin-engineering experiments.

As for the mutant G24N, the situation is slightly different. There are no apparent new interactions observed and the two fold increase in affinity is therefore difficult to rationalize. While enthalpic contribution is almost the same, the entropy term becomes less unfavourable (difference of about 3 kJ/mol), which could imply that the mutant protein shows better surface complementarity to the interacting sugar. The side chain of Asp 24 makes an additional hydrogen bond with carboxylic group of Asp96 and closes the binding pocket that therefore cannot accommodate any further water molecule that is seen in all four binding sites of the wild type protein in proximity of Asp96.

### Complexes of mutants with methyl α-D-mannopyranoside

**Figure 6:**
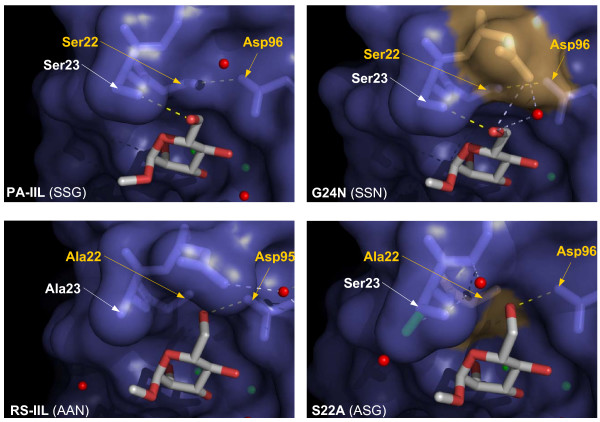
**Comparison of S22A/RSIIL and G24N/PA-IIL with Me-α-Man with important linkages highlighted**. A) PA-IIL, B) G24N, C) RS-IIL, D) S22A in a surface/stick representation. Mutated amino acids are in light brown. Interactions responsible for sugar preference are in yellow, newly created hydrogen bonds are in blue. Figure clearly demonstrates different orientation of O6 of methyl mannoside.

Crystal structures have been determined for the complexes of mutants S22A and G24N with Me-α-Man, allowing the precise description of the involvement of these two amino acids in the binding (Figure 6). In the case of the G24N mutant, the same order of sugar preference is observed as in the case of the wild type lectin. The total Gibbs energy is more favourable than in the case of the native lectin, though only slightly. The difference in thermodynamics of the interactions can be seen in the different ratio of the components. Whereas, in the case of the native protein, the entropic contribution is favourable and complements lower enthalpic contribution, the enthalpic part of the mutant ΔG differs by-9.3 kJ/mol from the wild type, and is compensated by an unfavourable entropic contribution, (nevertheless, the entropic contribution represents only 8% of the total Gibbs energy change).

The analysis of the crystal structure offers a possible explanation. The oxygen O6 of mannoside, forced to point out from the molecule by the presence of serine 22 (like in the case of the wild type protein), participates in a new hydrogen bond interaction with the amino group of the newly present Asn24. This results in a higher value of association constant. The amino group of Asn24 also makes a bridging interaction with Asp96 via the capture of one molecule of water. Although the crystal structure of the S23A mutant with mannoside is not available at the moment, the reasoning used in the cases of G24N and the wild-type lectin can be used also here for the explanation of difference in thermodynamic properties between fucoside- and mannoside-interaction.

**Figure 7:**
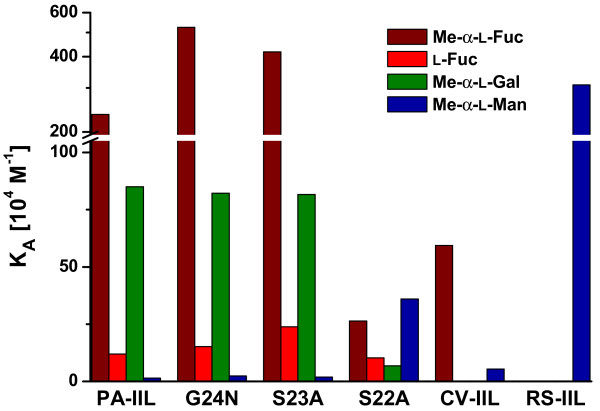
**Comparison of lectin binding affinities as determined by ITC**. Graphical representation of equilibrium association constants toward different monosaccharides for PA-IILwt [11, 14], PA-IIL mutants and their homologs from *C. violaceum *and *R. solanacearum *[18].

Last but not least, the structure of the S22A complex with mannoside was determined. This mutant is the only one of the three that displays a reversed order of preference between mannoside and fucoside and therefore the greatest change in thermodynamic characteristics. When comparing the mutant and the wild type protein, the enthalpic contribution for Me-α-Man binding is increased from -17.8 kJ/mol to -24.3 kJ/mol and the favourable entropic contribution TΔS from 5.9 kJ/mol to 7.4 kJ/mol, resulting in the total difference in free energy of -8.0 kJ/mol that corresponds to 25 times higher affinity of the mutant compared to the wild type protein. This demonstrates that the initial hypothesis of Ser22 holding the main key to affinity determination is correct. In the absence of the Ser22 hydroxyl group, there is no steric hindrance blocking the interaction of the sugar with Asp96. The crystal structure also reveals that Ser23, originally interacting with the mannoside O6, becomes more flexible. Up to three conformers can be observed in the binding site of S22A/Me-α-Man complex. Moreover, the complex closely resembles the previously determined crystal structure of the RS-IIL lectin/mannoside complex, whose characteristics have been previously described [16]. Overall, the S22A mutant can be described as both fucose- and mannose- binding. Similar features were recently described for the CV-IIL lectin from *C. violaceum*. It contains the Ser-Ala-Ala triad at positions 22, 23 and 24 and, both in terms of sequence and specificity, lies between PA-IIL and RS-IIL. CV-IIL prefers fucose but it is well adapted to bind mannose, showing affinity for Me-α-Man four times higher than PA-IIL [18]. A graphical representation of binding affinities for all mentioned lectins is shown in Figure 7.

## Conclusion

Understanding and rationalising the structural and thermodynamic basis of interaction between lectins from pathogens and carbohydrates opens the door to designing high affinity inhibitors. On the other hand, detailed depiction of the interaction and description of crucial amino acids for affinity and/or specificity of the lectins can lead to targeted protein-engineering of the carbohydrate-binding proteins and fine tuning their properties for biotechnology, pharmacology and bioanalytical applications. Mutagenesis of amino acids forming the specificity binding loop of the high affinity lectin from *P. aeruginosa *allowed the amino acids responsible for sugar preference to be determined. This knowledge could initiate further studies using a combination of rational site directed mutagenesis and directed evolution with the goal of preparing lectins with advanced and novel properties.

## Methods

### Site-directed mutagenesis and expression

A construct named pET-25(b+)pa2l, containing the plasmid pET-25(b+) (Novagen) and the full-length wild type *P. aeruginosa *PA-IIL encoding gene [10], was used as a template. Site-directed mutagenesis was performed with the QuikChange™ Site-Directed Mutagenesis Kit (Stratagene) following the manufacturer's instructions. The oligonucleotides used for the mutations were constructed as follows:

Mutant PA-IILS22A, primer pair PA-IILS22Asense/antisense (5'-CCG CCT TCG CCA ACG CGT CCG GAA CCC AGA CGG-3'/5-CCG TCT GGG TTC CGG ACG CGT TGG CGA AGG CGG-3'); mutant PA-IILS23A, primer pair PA-IILS23Asense/antisense (5'-CCG CCT TCG CCA ACT CGG CCG GAA CCC AGA CGG-3'/5'-CCG TCT GGG TTC CGG CCG AGT TGG CGA AGG CGG-3'); mutant PA-IILG24N, primer pair PA-IILG24Nsense/antisense (5'-CCG CCT TCG CCA ACT CGT CCA ACA CCC AGA CGG TGA ACG-3'/5'-CGT TCA CCG TCT GGG TGT TGG ACG AGT TGG CGA AGG-3'). Mutations were confirmed by DNA sequencing and new constructs were transformed into the *E. coli *Tuner(DE3) strain (Novagen).

### Purification of recombinant wild-type and mutant PA-IIL proteins

Wild type PA-IIL lectin was expressed in *E. coli *BL21(DE3) (Novagen) and purified on a mannose-agarose column as previously described [10]. PA-IIL mutants were prepared in similar way. *E. coli* Tuner (DE3) cells harbouring the plasmid encoding the desired mutant protein were grown at 37°C until OD_600 _= 0.6. After a three hour induction by 0.5 mM isopropylthiogalactoside at 30°C cells were harvested, disrupted by sonication and the supernatant applied to a D-mannose-agarose column (Sigma-Aldrich, USA) equilibrated by buffer composed of 20 mM Tris/HCl, 100 mM NaCl, 100 μM CaCl_2_, pH 7.5. After washing out unbound proteins, mutant proteins were eluted from the column using the same buffer containing 0.1 M D-mannose. Afterwards, the proteins were purified to homogeneity by the rapid, one step purification procedure previously described [10]. The purified proteins were dialysed against distilled water for 1 week (for the removal of D-mannose) and stored at -20°C in lyophilized form.

### Crystallization and structure determination of PA-IIL mutants

Mutants of PA-IIL were crystallised in complexes with fucose as their co-crystallisation with other sugars were not successful. The crystals were grown using the hanging drop vapour-diffusion method and conditions screened using the Hampton screens I and II. The best crystals obtained were soaked for 24 h in 10 mM Me-α-D-Man and Me-α-L-Fuc in the precipitant solution.

The different mutants gave co-crystals with fucose using the following conditions: 0.2 M ammonium sulphate, 30% PEG 4000 (G24N/Me-α-Fuc), 0.1 M HEPES (pH 7.5), 10 % isopropanol, 20% PEG 4000 (G24N/Me-α-Man), 0.2 M ammonium sulphate, 0.1 M Tris/HCl (pH 8.5), 30% PEG 4000 (S22A/Me-α-Man), 0.2 M lithium sulfate, 0.1 M Tris/HCl (pH 8.5), 30% PEG 4000 (S22A/Me-α-Fuc), 0.2 M calcium chloride, 0.1 M sodium acetate (pH 7.5), 20% isopropanol (S23A/Me-α-Fuc). Crystals, after soaking, were cryo-protected by soaking in 25 % glycerol v/v in the precipitant solution for as short a time as possible and were subsequently cryo-cooled to 100 K. All data images were recorded on beamlines ID14-2 or ID29 at the ESRF (Grenoble, France). The collected data were processed with MOSFLM [19] and scaled and converted to structure factors using the CCP4 program suite[20]. The phases were determined by the molecular replacement technique with the MOLREP program of CCP4 using the monomeric PA-IIL/Fuc structure (PDB code 1UZV) [12] stripped of water molecules, calcium ions and sugar ligands as the search probe. All complexes contain tetramers in the asymmetric units. Initial structures were entirely rebuilt automatically using ARP/warp [21] and refinement was performed with REFMAC [22].

The mutations were verified using *fo-fc *and *2fo-fc *electron density maps after molecular replacement using the native structure. Alternating cycles of refinement with manual construction using Coot [23] of missing residues, alternative conformations, water molecules, calcium ions and sugar ligands resulted in the final structures.

Coordinates and structure factors of the mutant complexes with Me-α-D-Man and Me-α-L-Fuc have been deposited in the Protein Data Bank under following codes: 2JDU (G24N/Me-α-Fuc), 2JDY (G24N/Me-α-Man), 2JDN (S22A/Me-α-Man), 2JDM (S22A/Me-α-Fuc) and 2JDP (S23A/Me-α-Fuc). Molecular drawings were prepared with PYMOL [24].

### ITC Analysis

Titration calorimetry experiments were performed using a Microcal (Northampton, MA) VP-ITC microcalorimeter. All titrations were made in 0.1 M Tris-HCl buffer containing 30 μM CaCl_2 _(pH 7.5) at 25°C. Aliquots of 10 μL of each carbohydrate, dissolved in the same buffer, were added at 8 min intervals to the lectin solution present in the calorimeter cell. In the titrations, the protein concentration in the cell varied from 0.06 to 0.2 mM. The sugar concentration in the 250 μl syringe was 10–20 times higher than the protein concentration used in the experiment (from 1.2 to 3.5 mM) The temperature of the cell was kept at 25 ± 0.1°C. Aliquots of 15 and 10 μl of the ligand solution were added in 8 min intervals to the lectin solution present in the calorimeter cell. Data from a control experiment performed via identical injections of monosaccharide into the cell containing only a buffer were subtracted prior to data analysis. Integrated heat effects were analyzed by nonlinear regression using a single site binding model (Microcal Origin 7). Fitted data yielded the association constant (K_a_) and the enthalpy of binding (ΔH). Other thermodynamic parameters, i.e., changes in free energy (ΔG) and entropy (ΔS), were calculated from the equation

ΔG = ΔH – TΔS = RT ln K_a_

where T is the absolute temperature and R = 8.314 J mol^-1 ^K^-1^. At least three independent titrations were performed for each ligand that was tested.

## Abbreviations

PA-IIL, lectin II from *Pseudomonas aeruginosa*, RS-IIL, mannose-binding lectin from *Ralstonia solanacearum*; CV-IIL, fucose-binding lectin from *Chromobacterium violaceum*; CF, cystic fibrosis; L-Gal, L-galactose; Fuc, L-fucose; Man, D-mannose; Me-α-L-Gal, methyl α-L-galactopyranoside; Me-α-Fuc, methyl α-L-fucopyranoside; Me-α-Man, methyl α-D-mannopyranoside; S22A, S23A and G24N, PA-IIL lectin mutated on positions 22, 23 and 24

## Authors' contributions

JA carried out microcalorimetry measurements and drafted manuscript. MPconstructed all mutants, purified and crystallized proteins. CS carried out x-ray data collection, structures resolution and refinement. EPM supervised crystallography and corrected the manuscript. AI participated in study design and coordination and corrected the manuscript. MW conceived the study and participated in its design and coordination. All authors read and approved the final manuscript.
